# Interferon‐regulated suprabasin is essential for stress‐induced stem‐like cell conversion and therapy resistance of human malignancies

**DOI:** 10.1002/1878-0261.12480

**Published:** 2019-06-10

**Authors:** Sona Hubackova, Miroslav Pribyl, Lenka Kyjacova, Alena Moudra, Rastislav Dzijak, Barbora Salovska, Hynek Strnad, Vojtech Tambor, Terezie Imrichova, Jiri Svec, Pavel Vodicka, Radka Vaclavikova, Lukas Rob, Jiri Bartek, Zdenek Hodny

**Affiliations:** ^1^ Laboratory of Genome Integrity Institute of Molecular Genetics of the ASCR, v. v. i. Prague Czech Republic; ^2^ Molecular Therapy Group Institute of Biotechnology Czech Academy of Sciences BIOCEV Vestec, Prague‐West Czech Republic; ^3^ Laboratory of Genomics and Bioinformatics Institute of Molecular Genetics of the ASCR, v. v. i. Prague Czech Republic; ^4^ Biomedical Research Center University Hospital Hradec Kralove Czech Republic; ^5^ Laboratory of Cell and Developmental Biology Institute of Molecular Genetics of the ASCR, v. v. i. Prague Czech Republic; ^6^ Department of Radiotherapy and Oncology Third Faculty of Medicine Charles University Prague Czech Republic; ^7^ Department of the Molecular Biology of Cancer Institute of Experimental Medicine Academy of Sciences of the Czech Republic Prague Czech Republic; ^8^ Institute of Biology and Medical Genetics 1st Medical Faculty Charles University Prague Czech Republic; ^9^ Laboratory of Pharmacogenomics Biomedical Centre Faculty of Medicine in Pilsen Charles University Pilsen Czech Republic; ^10^ Department of Gynecology and Obstetrics Third Faculty of Medicine Vinohrady University Hospital Charles University Prague Czech Republic; ^11^ Danish Cancer Society Research Center Copenhagen Denmark; ^12^ Department of Medical Biochemistry and Biophysics Division of Genome Biology Science for Life Laboratory Karolinska Institute Stockholm Sweden; ^13^ Present address: Centre for Biomedicine and Medical Technology Mannheim Medical Faculty Mannheim University Heidelberg Mannheim Germany

**Keywords:** 5‐azacytidine, cancer stem‐like cells, interferon response, suprabasin, therapy‐resistance

## Abstract

Radiation and chemotherapy represent standard‐of‐care cancer treatments. However, most patients eventually experience tumour recurrence, treatment failure and metastatic dissemination with fatal consequences. To elucidate the molecular mechanisms of resistance to radio‐ and chemotherapy, we exposed human cancer cell lines (HeLa, MCF‐7 and DU145) to clinically relevant doses of 5‐azacytidine or ionizing radiation and compared the transcript profiles of all surviving cell subpopulations, including low‐adherent stem‐like cells. Stress‐mobilized low‐adherent cell fractions differed from other survivors in terms of deregulation of hundreds of genes, including those involved in interferon response. Exposure of cancer cells to interferon‐gamma but not interferon‐beta resulted in the development of a heterogeneous, low‐adherent fraction comprising not only apoptotic/necrotic cells but also live cells exhibiting active Notch signalling and expressing stem‐cell markers. Chemical inhibition of mitogen‐activated protein kinase/ERK kinase (MEK) or siRNA‐mediated knockdown of extracellular signal‐regulated kinase 1/2 (Erk1/2) and interferon responsible factor 1 (IRF1) prevented mobilization of the surviving low‐adherent population, indicating that interferon‐gamma‐mediated loss of adhesion and anoikis resistance required an active Erk pathway interlinked with interferon signalling by transcription factor IRF1. Notably, a skin‐specific protein suprabasin (SBSN), a recently identified oncoprotein, was among the top scoring genes upregulated in surviving low‐adherent cancer cells induced by 5‐azacytidine or irradiation. *SBSN* expression required the activity of the MEK/Erk pathway, and siRNA‐mediated knockdown of *SBSN* suppressed the low‐adherent fraction in irradiated, interferon‐gamma‐ and 5‐azacytidine‐treated cells, respectively, implicating *SBSN* in genotoxic stress‐induced phenotypic plasticity and stress resistance. Importantly, *SBSN* expression was observed in human clinical specimens of colon and ovarian carcinomas, as well as in circulating tumour cells and metastases of the 4T1 mouse model. The association of *SBSN* expression with progressive stages of cancer development indicates its role in cancer evolution and therapy resistance.

**Abbreviations**
5‐AC: 5‐aza‐2′‐deoxycytidine
Akt: protein kinase B
Bcl XL: B‐cell lymphoma‐extra large
Bim: Bcl‐2‐like protein 11
CSCs: cancer stem cells
DLL1: delta‐like protein 1
DLL4: delta‐like protein 4
EMT: epithelial‐to‐mesenchymal transition
Erk: extracellular signal‐regulated kinase
ESCC: oesophageal squamous cell carcinoma
fIR: fractionated irradiation
GAPDH: glyceraldehyde 3‐phosphate dehydrogenase
GOBP: gene ontology biological process
IFNβ: interferon beta
IFNγ: interferon gamma
IRF1: interferon responsible factor 1
IRF7: interferon responsible factor 7
ISG15: interferon‐stimulated gene 15
LFC: log2 fold change
MAPK: mitogen‐activated protein kinase
MEK: MAPK/ERK kinase
MX1: MX dynamin‐like GTPase 1
NF‐κB: nuclear factor κB
OCT4: octamer‐binding transcription factor 4
PCa: prostate cancer
SBSN: suprabasin
SILAC: stable isotope labelling with amino acids in cell culture
SOCS1: suppressor of cytokine signalling 1
Sox2: SRY (sex determining region Y)‐box 2
TNF: tumour necrosis factor

## 1 Introduction

Over the last decades, genotoxic therapies including chemo‐ or radiotherapy have been the prevailing modalities used to treat cancer patients. Unfortunately, the initial effectiveness of radiotherapy and chemotherapy is commonly undermined by the primary and/or acquired resistance of tumour cells associated with the development of recurrent disease and progression towards a largely incurable metastatic stage that is responsible for over 90% of cancer‐related mortality. The mechanisms underlying the occurrence of cancer resistance to radiotherapy and chemotherapy are complex and still poorly understood. Accumulating evidence indicates that the resistance of cancer cells to radiotherapy and chemotherapy encompasses several different but partly interlinked mechanisms comprising therapy‐induced molecular programs together with the initial intra‐tumour genetic and epigenetic heterogeneity (Burrell *et al*., 2013; Kreso *et al*., 2013; Swanton, 2012).

Notably, recent research has provided evidence that the cellular response to genotoxic stress can contribute to phenotypic reprogramming of cancer cells into more resistant phenotypes. Such process commonly involves induction of epithelial‐to‐mesenchymal transition (EMT) and occurrence of cancer cells with stem‐like characteristics (Ghisolfi *et al*., 2012; Gomez‐Casal *et al*., 2013; Kyjacova *et al*., 2015; Skvortsova *et al*., 2008). Generally, cancer‐induced mesenchymal and stem‐like cells appear to be more resistant to genotoxic stress compared with epithelial cancer cells (Blanpain *et al*., 2011). In addition, genotoxic therapies increase the migratory and invasive properties of malignant cells (Moncharmont *et al*., 2014) and alter the tumour‐associated microenvironment, collectively promoting metastatic behaviour of treatment‐surviving cancer cells (Ruegg *et al*., 2011).

There is mounting evidence that EMT is coupled with activation of the stem cell programme in both normal (Mani *et al*., 2008) and transformed cells (Han *et al*., 2013; Rhim *et al*., 2012). Whereas cancer stem cells (CSC) express EMT transcription factors such as Twist1, Snail and Slug, in contrast, EMT‐undergoing cells were found enriched for CSC markers (Thiery *et al*., 2009). Related to the non‐responsiveness of CSC to the currently used treatment modalities, some authors detected putative CSC following the therapy as the only residual cell population (Levina *et al*., 2008).

Suprabasin (SBSN) was originally found in the suprabasal layer of murine and human keratinocytes and was described as an epidermal differentiation marker and component of keratinocyte cornified envelope and as a substrate for transglutaminase‐mediated cross‐linking (Alam *et al*., 2014; Park *et al*., 2002). However, its significant upregulation after increased demethylation of CpG islands in promoter of non‐small cell lung carcinoma (Glazer *et al*., 2009) and salivary gland adenoid cystic carcinoma has been described (Shao *et al*., 2012), and later experiments revealed a role of *SBSN* in tumour cell proliferation and invasiveness (Zhu *et al*., 2016). These studies collectively suggest that *SBSN* is a new candidate oncogene contributing to tumour development and progression. Although Brother of the Regulator of Imprinted Sites factor has been found to contribute to regulation of *SBSN* expression (Gaykalova *et al*., 2012), detailed mechanistic insights into regulation of *SBSN* expression in tumours is still missing.

Recently, we have shown that exposure of human metastasis‐derived prostate cancer (PCa) cell lines to a clinically relevant fractioned ionizing radiation (fIR) regimen resulted in development of three distinct fIR‐surviving populations *in vitro* (Kyjacova *et al*., 2015): (1) senescent‐like adherent cells with the potential to restart proliferation after the last dose of IR; (2) low‐adherent dormant cells with stem‐like cell traits and preserved competence to re‐attach and restore proliferation (low‐adherent cells); and (3) re‐adherent cells derived from the low‐adherent fraction with reverted epithelial features and retained tumourigenicity supporting a model of bidirectional interconversion in establishment of tumour cell heterogeneity (reviewed in van Neerven *et al*., 2016). We observed the involvement of an EMT‐like programme in the response of PCa cells to fIR with such a phenotypic switch strongly dependent on the extracellular signal‐regulated kinase 1/2 (Erk1/2)‐mediated expression of Snail and associated with repression of E‐cadherin. However, the basis for the heterogeneous response of cancer cells to genotoxic stress remains poorly understood.

In this study, we explored the signalling pathways involved in phenotypic remodelling of cancer cells induced by radiotherapy and/or chemotherapy. Comparing transcription and proteomic profiles of three human cancer models of interconverting subpopulations of stress‐surviving tumour cells we identified a hierarchy of interferon and Erk signalling pathways in the regulation of *SBSN* and its novel function in establishment of the phenotypic plasticity and heterogeneity of therapy‐resistant cancer cell populations.

## 2 Materials and methods

### 2.1 Chemicals and antibodies

Acetone, PMA (phorbol 12‐ myristate 13‐ acetate), Ponceau S solution, 4′,6‐diamidino‐2‐phenylindole (DAPI), mitogen‐activated protein kinase (MAPK)/ERK kinase (MEK) inhibitor U0126, tumour growth factor beta receptor 1 inhibitor SB431542 and 5‐azacytidine (5‐AC) were purchased from Sigma (St. Louis, MO, USA). Human interferon gamma (IFNγ) and human interferon beta (IFNβ) were purchased from Peprotech (Rocky Hill, NJ, USA). MEK kinase inhibitor selumetinib (AZD6244) was purchased from Selleckchem (Munich, Germany).

For immunoblotting and indirect immunofluorescence, the following antibodies were used: rabbit monoclonal antibodies against Snail, B‐cell lymphoma‐extra large (Bcl‐XL), Bcl‐2‐like protein 11 (Bim), protein kinase B (Akt), Akt phosphorylated on serine 473, rabbit polyclonal antibodies against β‐actin, Bim, SMAD2 phosphorylated on serine 465/467, STAT1 phosphorylated on tyrosine 701, Erk1 and mouse monoclonal Erk 1/2 (all from Cell Signaling Technology, Danvers, MA, USA), mouse monoclonal antibody against glyceraldehyde 3‐phosphate dehydrogenase (GAPDH; GeneTEX, Irvine, CA, USA), mouse monoclonal antibodies against E‐cadherin and integrin α‐2 (ITGA2; BD Biosciences, San Jose, CA, USA), rabbit polyclonal antibody against phosphothreonine 202/phosphotyrosine 204 of Erk1/2 (Promega, San Luis Obispo, CA, USA), rabbit polyclonal antibody against *SBSN* (Abgent, San Diego, CA, USA), rabbit polyclonal antibody against *SBSN* (Sigma), rabbit polyclonal antibodies against Erk2, interferon responsible factors 1 and 7 (IRF1 and IRF7; all from Santa Cruz, Dallas, TX, USA). Mouse monoclonal antibody against γ‐tubulin was provided by Pavel Draber (Institute of Molecular Genetics, Prague, Czech Republic). IgG‐HRP anti‐rabbit (170–6515) and anti‐mouse (170–6516) secondary antibodies produced in goat were purchased from Bio‐Rad Laboratories (Hercules, CA, USA). Alexa Fluor 488 anti‐rabbit was purchased from Thermo Fisher Scientific (Waltham, MA, USA). Primary antibodies for immunoblotting and indirect immunofluorescence were diluted 1 : 1000 (1 : 5000 for GAPDH) in 2.5% non‐fat milk and 1 : 100 in 10% FBS/1 × PBS, respectively. Secondary antibodies for immunoblotting and indirect immunofluorescence were diluted 1 : 10 000 in 2.5% non‐fat milk and 1 : 1200 in 10% FBS/1 × PBS, respectively.

### 2.2 Cell cultures

All cell lines were obtained from American Type Culture Collection (Manassas, VA, USA). Human embryonic kidney cells HEK293, cervix adenocarcinoma HeLa, prostate carcinoma DU145, glioblastoma U373 and breast carcinoma MCF‐7 cell lines were cultured in Dulbecco's modified Eagle's medium containing 4.5 g·L^−1^ glucose (Biochrom, Berlin, Germany). HPV‐16 E6/E7‐transformed human mesenchymal stem cell line HS‐5 and ovarian carcinoma SK‐OV‐3 cell lines were cultured in RPMI. All culture media were supplemented with 10% FBS (Gibco, Carlsbad, CA, USA), 100 U·mL^−1^ penicillin and 100 μg·mL^−1^ streptomycin sulphate (Sigma). RPMI medium was in addition supplemented with 1% non‐essential amino acids (Sigma‐Aldrich). Cells were kept at 37 °C at 5% CO_2_ atmosphere and 95% humidity.

DU145, MCF‐7 and U373 cells were irradiated 24 h after seeding (22 000 cells·cm^−2^) with orthovoltage X‐ray instrument T‐200 (Wolf‐Medizintechnik, St. Gangloff, Germany) using 0.5 Gy·min^−1^ dose rate and thorium filter either daily with 10 doses 2 Gy or with a single dose of 2 or 10 Gy (Kyjacova *et al*., 2015). Half volume of culture medium was changed every 48 h for a fresh one. All low‐adherent cells were collected by centrifugation (300 ***g*** for 10 min) and returned back into cultivation flask. Twenty‐four hours after the last dose of fIR and 72 h after a single dose, low‐adherent and adherent cells were harvested separately.

**Figure 1:**
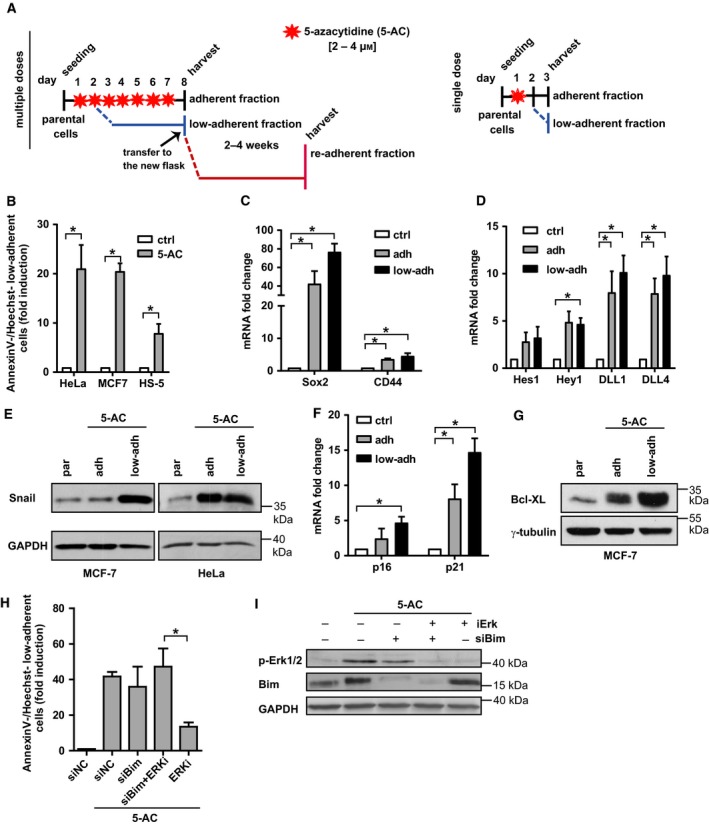
(A) Schematic representation of the treatment protocol using either multiple doses of 5‐AC every 24 h (left) of a single dose of 5‐AC (right), resulting in generation of chemoresistant populations (adherent, low‐adherent and re‐adherent) in human HeLa and MCF‐7 cancer cell lines or HS‐5 cells. (B) Loss of adhesion expressed as a ratio (fold induction) of Annexin V^−^/Hoechst^−^ detached cells relative to all Annexin V^−^/Hoechst^−^ cells (see Section 2 Materials and methods) assessed by FACS in control cells or cells treated with 2 μm 5‐AC for 7 days. (C) RT‐qPCR quantification of Sox2 and CD44 mRNA levels in parental, adherent and low‐adherent HeLa cells treated with 4 μm 5‐AC for 7 days. (D) RT‐qPCR quantification of *DLL*1, *DLL*4, Hes1 and *Hey1* mRNA levels in parental, adherent and low‐adherent HeLa cells treated with 4 μm 5‐AC for 7 days. (E) Immunoblotting detection of Snail in parental, adherent and low‐adherent HeLa and MCF‐7 cells treated with 4 μm 5‐AC for 72 h. GAPDH was used as a loading control. (F) RT‐qPCR quantification of p16 and p21 mRNA levels in parental, adherent and low‐adherent HeLa cells treated with 2 μm 5‐AC for 72 h. (G) Immunoblotting detection of Bcl‐XL in parental, adherent and low‐adherent HeLa cells treated with 4 μm 5‐AC for 72 h. Gamma‐tubulin was used as a loading control. (H) Estimation of low‐adherent fraction after inhibition of Erk kinase pathway with selumetinib (ERKi, 1 μm) in combination with siRNA knockdown of *Bim* (siBim) expressed as a ratio (in fold induction) of Annexin V^−^/Hoechst^−^ detached cells relative to all Annexin V^−^/Hoechst^−^ cells assessed by FACS in HeLa cells treated with 2 μm 5‐AC for 72 h. Non‐targeting siRNA (siNC) was used as a control. (I) Immunoblotting detection of Erk phosphorylated on threonine 202/tyrosine 204 and Bim in HeLa cells treated with 2 μm 5‐AC for 72 h after knockdown of *Bim* (siBim) or in the presence of MEK/Erk inhibitor selumetinib (ERKi; 1 μm). GAPDH was used as a loading control. Data are shown as mean values ± SEM, with *n *=* *3 per group. Two‐way ANOVA was used for multigroup comparisons followed by Tukey's *post hoc* test. The asterisk represents *P* < 0.05.

To treat cells with 5‐AC, HeLa, HS‐5 and MCF‐7 cells were seeded at a density of 22 000 cells·cm^−2^ 24 h before 5‐AC addition. A stock solution of 5‐AC in sterile water was added either directly into culture medium (day 1, 3, 5, 7) or with exchange of a half volume of culture medium (day 2, 4 and 6) to a final concentration 2 or 4 μm with or without MEK inhibitor selumetinib (1 μm). Low‐adherent cells were always re‐collected as in the case of fIR. Low‐adherent and adherent cells were harvested separately at day 8. Alternatively, a single dose of 5‐AC was used and both low‐adherent and adherent cells were harvested 72 h later (see Fig. 1A).

To treat cells with interferons, HeLa or MCF‐7 cells were seeded at a density of 22 000 cells·cm^−2^. Next day the culture medium was exchanged for a fresh one with or without IFNγ (5 ng·mL^−1^) or IFNβ (50 ng·mL^−1^) and cells were incubated for an additional 3 (HeLa) or 6 days (MCF‐7). In the case of Erk pathway inhibition, MEK inhibitor selumetinib (1 μm) was added together with the first addition of IFNγ and then every 24 h either directly to culture medium or with medium exchange (half volume) made every 48 h with re‐collection of all low‐adherent cells as described above.

### 2.3 Human colon carcinoma and ovarian cancer samples

See Table S3 for patient characteristics. Informed written consent has been obtained from all patients in compliance with the Declaration of Helsinki. The study was approved by the Ethics Committee of the Third Faculty of Medicine, Charles University, Prague, Ethical Commission of the National Institute of Public Health in Prague and by the Ethics committee of the General University Hospital Prague.

### 2.4 4T1 mouse metastatic model

Six‐week‐old BALB/c mice (females) were injected with 1 × 10^6^ 4T1 cells in PBS into mammary gland. Mice were sacrificed after 3 weeks when tumours on average reached ± 800 mm^3^; lungs, liver and blood were removed and processed according to protocol described by Pulaski *et al*. (2001). To determine the level of *SBSN* expression, 4T1 colonies selected by 6‐thioguanine were harvested and processed as described below in section on Quantitative real time PCR. Experiments were approved by the Czech Academy of Sciences Ethics Committee and performed according to the Czech Council guidelines for the Care and Use of Animals in Research and Teaching.

### 2.5 Colony‐forming assay

To estimate the potential of low‐adherent cells to reestablish adherence, the modified colony‐forming assay was used as reported (Kyjacova *et al*., 2015). Briefly, 3 days after the exposure to treatment, low‐adherent cells were transferred onto a new plate and left for 24 h to allow adherence of residual mitotic cells. The remaining low‐adherent cells were transferred to a new plate and allowed to re‐adhere for 24–30 days. To visualize formation of colonies, cells were fixed at −20 °C with 96% ice‐cold methanol for 10 min and then stained with 0.5% crystal violet in ethanol at room temperature for 5 min.

### 2.6 Indirect immunofluorescence

U373 cells were transfected by siRNA 24 h after seeding at a density of 22 000 cells·cm^−2^ on glass coverslips. Culture medium was changed 24 h after transfection. Cells were irradiated by a single dose of 10 Gy 48 h post‐transfection. Seventy‐two hours post‐irradiation, cells were fixed with 4% formaldehyde for 15 min and then permeabilized by 0.1% Triton X‐100. After washing with PBS, cells were incubated in 10% FBS/1 × PBS for 60 min, with primary antibody at room temperature for 1 h, and after PBS wash with secondary antibodies at room temperature for 1 h. After washing with 1 × PBS and ddH_2_O, cells were stained with 1 μg·mL^−1^ DAPI (Sigma), washed with ddH_2_O and mounted with ProLong™ Gold Antifade Mountant (Thermo Fisher Scientific). Images were acquired by a Leica DM6000 fluorescence microscope (Leica Microsystems, Wetzlar, Germany).

Low‐adherent MCF‐7 cells were generated using a single dose of 4 μm 5‐AC. After 5 weeks with medium exchange every 3–4 days, cells were washed with PBS and cytospin onto microscopic slides (400 ***g*** for 5 min; Centurion Scientific, K3 Series, Irvine, CA, USA). Attached cells were fixed using 4% formaldehyde for 15 min and then permeabilized by 0.1% Triton X‐100. After washing with PBS and ddH_2_O, cells were stained with 1 μg·mL^−1^ DAPI (Sigma), washed with ddH_2_O and mounted with ProLong™ Gold Antifade Mountant (Thermo Fisher Scientific). Images were acquired with a Leica TCS SP8 confocal laser microscopy system (Leica Microsystems).

### 2.7 Magnetic‐activated cell sorting (MACS)

Cells were treated for 72 h and low‐adherent cells were collected 24 h after the last dose of the particular treatment. The Annexin V‐negative fraction of low‐adherent human cancer cells was obtained by incubation of low‐adherent cells with Dead Cell Removal MicroBeads (Dead Cell Removal Kit, Miltenyi Biotec, Bergisch Gladbach, Germany) for 15 min and separated using the magnetic field of an AutoMACS Pro magnetic separator (Miltenyi Biotec). The separated cells were processed immediately for immunoblotting, qRT‐PCR or whole genome expression analysis.

### 2.8 Detection of apoptosis using fluorescence‐activated cell sorting (FACS)

The numbers of viable and dead cells were analyzed using an LSRII flow cytometer (BD Biosciences). Cells (adherent in one vial and dead plus low‐adherent in other vial) were collected (400 ***g*** at 4 °C for 5 min), washed with PBS and stained for 15 min at room temperature with 0.3 μL of Annexin V‐Dyomics 647 in 100 μL of 1 × Annexin V binding buffer (Apronex, Prague, Czech Republic). Immediately before analysis, 0.3 μL of Hoechst 33258 (10 mm; Invitrogen, Carlsbad, CA, USA) was added. The percentage of low‐adherent cells (Annexin V^−^/Hoechst^−^) was calculated according to the formula low‐adherent % = [(low‐adherent cells/(low‐adherent cells + live‐adherent cells)]*100. The calculated percentages of surviving low‐adherent cells after the treatments were further normalized to the percentage of low‐adherent cells in control conditions.

### 2.9 SDS/PAGE and immunoblotting

Cells were washed twice with PBS, harvested into Laemmli SDS sample lysis buffer (2% SDS, 50 mm Tris‐Cl, 10% glycerol in double‐distilled H_2_O) and sonicated (3 × 15 s at 4 μm amplitude with 15‐s cooling intervals) using Soniprep 150 (MSE, London, UK). Conditioned culture media were precipitated by acetone overnight and samples then centrifuged (4000 ***g***, 10 min), and air‐dried pellets were dissolved into Laemmli SDS sample lysis buffer and sonicated (3 × 15 s at 4 μm amplitude with 15‐s cooling intervals). Concentration of proteins was estimated by the BCA method (Pierce Biotechnology, Rockford, IL, USA). DTT 100 mm and 0.01% bromophenol blue were added to cell lysates before separation by SDS/PAGE. The same amount of protein (35–50 μg) was loaded into each well. Proteins were electrotransferred onto a nitrocellulose membrane using wet transfer and detected by specific antibodies combined with horseradish peroxidase‐conjugated secondary antibodies (goat anti‐rabbit, goat anti‐mouse; Bio‐Rad). Peroxidase activity was detected by ECL (Pierce Biotechnology) or SuperSignal West Dura Extended Duration Substrate (Thermo Fisher Scientific). GAPDH, γ‐tubulin or β‐actin was used as a marker of equal loading.

### 2.10 RNA interference

Specific siRNA was introduced into cells using Lipofectamine RNAiMAX (Invitrogen) following the manufacturer's instructions. Forty‐eight hours after lipofection, cells were treated with fIR/IR, IFNγ and 5‐AC. siRNA was purchased from Thermo Fisher Scientific or Dharmacon (Lafayette, CO, USA). Non‐targeting siRNA sequences (Silencer® Select Negative Control No. 1, #4390843, Thermo Fisher Scientific, , Waltham, MA, USA) were used as negative control siRNA (siNC). Sense sequences of used siRNAs are listed below: siErk1#1: 5′‐GGA CCG GAU GUU AAC CUU Utt‐3′, siErk1#2: 5′‐UGA UGG AGA CUG ACC UGU Att‐3′, siErk2#1: 5′‐CAA CCA UCG AGC AAA UGA Att‐3′, siErk2#2: 5′‐GCA GAA AUG CUU UCU AAC Att‐3′, si*SBSN*#1: 5′‐AGA AGG UCA UUG AAG GGA Utt‐3′, si*SBSN*#2: 5′‐GGA GAA GGU UUU CAA CGG Att‐3′, siIRF1#1: 5′‐GCUCAGCUGUGCGAGUGUA‐3′, siIRF1#2: 5′‐CCU CUG AAG CUA CAA CAG Att‐3′.

### 2.11 Quantitative real time PCR (RT‐qPCR)

Total RNA samples were isolated using RNeasy Mini Kit (Qiagen Sciences, Germantown, MD, USA) as described (Vlasakova *et al*., 2007). First strand cDNA was synthesized from 500 ng of total RNA with random hexamer primers using a High‐Capacity cDNA Reverse Transcription kit (Applied Biosystems, Foster City, CA, USA). RT‐qPCR was performed in ABI Prism 7300 (Applied Biosystems) using SYBR Select Master Mix containing SYBR GreenE dye (Applied Biosystems). The relative quantity of cDNA was estimated by the ΔΔCT method and data were normalized to RPL37a or β‐actin (ACTB).

The following primers were purchased from Eastport (Prague, Czech Republic): h*SBSN*: 5′‐CAG GCT GGA AAG GAA GTG GAG A‐3′, 5′‐CTT GAT GGC TGG AAG ATC CGC T‐3′; Snail (SNAI1): 5′‐TGC CCT CAA GAT GCA CAT CCG A‐3′, 5′‐GGG ACA GGA GAA GGG CTT CTC‐3′; hSox2: 5′‐CAA GAT GCA CAA CTC GGA GA‐3′, 5′‐GCT TAG CCT CGT CGA TGA AC‐3′; CD44: 5′‐CCA GAA GGA ACA GTG GTT TGG C‐3′, 5′‐ACT GTC CTC TGG GCT TGG TGT T‐3′; p16INK4A (CDKN2A): 5′‐CTC GTG CTG ATG CTA CTG AGG A‐3′, 5′‐GGT CGG CGC AGT TGG GCT CC‐3′; p21WAF1 (CDKN1A): 5′‐TCA CTG TCT TGT ACC CTT GTG C‐3′, 5′‐GGC GTT TGG AGT GGT AGA AA‐3′; delta‐like protein 1 (DLL1): 5′‐TGC CTG GAT GTG ATG AGC A‐3′, 5′‐ACA GCC TGG ATA GCG GAT ACA C‐3′; delta‐like protein 4 (DLL4): 5′‐CTG CGA GAA AGT GGA CAG G‐3′, 5′‐ACA GTC GCT GAC GTG GAG TTC A‐3′; Hes1: 5′‐GGA AAT GAC AGT GAA GCA CCT CC‐3′, 5′‐GAA GCG GGT CAC CTC GTT CAT G‐3′; Hey1: 5′‐TGT CTG AGC TGA GAA GGC TGG T‐3′, 5′‐TTC AGG TGA TCC ACG GTC ATC TG‐3′ RPL37a: 5′‐AGG AAC CAC AGT GCC AGA TCC‐3′, 5′‐ATT GAA ATC AGC CAG CAC GC‐3′; octamer‐binding transcription factor 4 (OCT4): 5′‐CAG CTT GGG CTC GAG AAG‐3′, 5′‐CCT CTC GTT GTG CAT AGT CG‐3′; β‐actin (ACTB): 5′‐CCA ACC GCG AGA TGA‐3′, 5′‐CCA GAG GCG TAC AGG GAT AG‐3′; Herc5: 5′‐CAA CTG GGA GAG CCT TGT GGT T‐3′, 5′‐CTG GAC CAG TTT GCT GAA AGT GG‐3′; suppressor of cytokine signalling 1 (SOCS1): 5′‐TTC GCC CTT AGC GTG AAG ATG G‐3′, 5′‐TAG TGC TCC AGC TCG AAG A‐3′; IRF1: 5′‐CTG GCA CAT CCC AGT GGA A‐3′, 5′‐CAT CCT CAT CTG TTG TAG CTT CAG A‐3′; IRF7: 5′‐CCA CGC TAT ACC ATC TAC CTG G‐3′, 5′‐GCT GCT ATC CAG GGA AGA CAC A‐3′; interferon‐stimulated gene 15 (ISG15): 5′‐CTC TGA GCA TCC TGG TGA GGA A‐3′, 5′‐AAG GTC AGC CAG AAC AGG TCG T‐3′; MX dynamin‐like GTPase 1 (MX1): 5′‐CTC CCA CTC CCT GAA ATC TG‐3′, 5′‐GAG CTG TTC TCC TGC ACC TC‐3′; m*SBSN*: 5′‐GCC AGG AAA TGA ACA GGT TGC AG‐3′, 5′‐TAC CCG CCT TGA CCT TGA G‐3′; mSox2: 5′‐TTT GTC CGA GAC CGA GAA GC‐3′, 5′‐CTC CGG GAA GCG TGT ACT TA‐3′.

The data are expressed as the means ± SEM of a minimum of three independent experiments performed in triplicate. The *P‐*values were estimated using two‐tailed Student's *t*‐test. *P*‐values < 0.05 were considered statistically significant.

### 2.12 Preparation of *SBSN* promoter‐driven luciferase construct

The *SBSN* proximal promoter‐driven luciferase reporter vector (pGL3‐*SBSN*) was prepared by cloning a 2071‐bp‐long proximal promoter region upstream of the *SBSN* gene initiation codon (GRCh38.p12 ch19: 35 528 282–35 530 351) into a pGL3‐Basic vector (Promega) utilizing Gibson assembly. For this purpose, two pairs of primers were used: (1) pGL3‐Basic FWD, 5′‐TTC CCG ACC TTC CCA GCA ATC TCG AGA TCT GCG ATC TAA GTA AGC T‐3′, pGL3‐Basic REV: 5′‐TTT TGG AGG GAG AGT CTC ACC CCG GGC TAG CAC GCG TAA G‐3′; and (2) *SBSN* 2071 bp upstream FWD: 5′‐CTT ACG CGT GCT AGC CCG GGG TGA GAC TCT CCC TCC AAA A‐3′, *SBSN* 2071 bp upstream REV: 5′‐CTT AGA TCG CAG ATC TCG AGA TTG CTG GGA AGG TCG GGA A‐3′. As templates for PCR, pGL3‐Basic vector or MCF‐7 genomic DNA isolated using Quick‐DNA isolation kit (Zymo Research Corp., Irvine, CA, USA) were used. Generated amplicons subsequently underwent multistep enzymatic reaction of Phusion DNA polymerase (Thermo Fisher Scientific), T5 exonuclease (New England Biolabs, Beverly, MA, USA) and *Taq* DNA ligase (New England Biolabs). Sequencing control was performed using two primers, LucNrev primer: 5′–CCT TAT GCA GTT GCT CTC C‐3′ and RVprimer3: 5′‐CTA GCA AAA TAG GCT GTC CC‐3′.

### 2.13 Transient transfection and dual‐luciferase reporter assay

HEK293 were seeded at a density of 22 000 cells·cm^−2^ onto a 60‐mm dish. Twenty‐four hours later the medium was exchanged and the cells were co‐transfected with 6 μg pGL3‐*SBSN*, pGL‐3‐Control (SV40 promoter) or pGL3‐Basic (promoterless) vector encoding *Firefly* luciferase together with 6 μg pRL‐TK vector encoding *Renilla* luciferase using the calcium phosphate method. After medium exchange 24 h post‐transfection, cells were treated with IFNγ (5 ng·mL^−1^) or PMA (20 nm). MEK inhibitor selumetinib (1 μm) was added to cell cultures either alone or simultaneously with addition of IFNγ. Samples were collected 48 h after treatments into 1 × Passive lysis buffer and further processed according to manufacturer's protocol (Promega). Luciferase activity was detected by 2104 EnVision Multimode plate reader (Perkin Elmer, Waltham, Massachusetts, USA). Luminometric data of *Firefly* luciferase activity were normalized to *Renilla* luciferase activity and protein concentration determined by BCA assay (Thermo Fisher Scientific).

### 2.14 Whole‐genome expression array

To obtain fIR‐resistant cell fractions (10 × 2 Gy daily), DU145 and MCF‐7 cells were seeded at a density of 14 000 cells·cm^−2^ into two 175‐cm^2^ cultivation flasks 24 h before first dose. Low‐adherent cells were recollected every medium exchange (48 h). Low‐adherent cells were separated from adherent ones 24 h after the last dose. One half of low‐adherent cells was harvested (low‐adherent fraction; see below) in parallel with adherent cells (adherent fraction) and the remaining low‐adherent cells were kept in culture to form re‐adherent colonies and harvested after 21 days (re‐adherent fraction). Proliferating cells served as controls (parental cells).

To generate 5‐AC‐resistant cell fractions (seven times the daily dose of 5‐AC), HeLa cells were seeded at a density of 14 000 cells·cm^−2^ into two 175‐cm^2^ cultivation flasks 24 h before first addition of 5‐AC (4 μm) and cultured as described above. Twenty‐four hours after the last dose of 5‐AC, half of low‐adherent cells was harvested in parallel with adherent cells. Re‐adherent cells were prepared in a similar manner as those for fIR. Proliferating cells served as control (parental fraction).

Annexin V‐negative (live) low‐adherent cells were separated from dead cells by MACS as described above. Total RNA of all cell fractions (parental, low‐adherent, adherent and re‐adherent) was isolated using RNeasy Mini Kit (Qiagen Sciences). RNA integrity was assessed in Agilent 2100 Bioanalyzer and RNA 6000 Nano LabChip (Agilent Technologies, Santa Clara, CA, USA). Total RNA was amplified using Illumina TotalPrep RNA Amplification Kit from Applied Biosystems according to the standard protocol, from a starting amount of 200 ng. The cDNA quality and quantity were assessed in Agilent 2100 Bioanalyzer and RNA 6000 Nano LabChip. The cDNA (750 ng) was hybridized, washed and scanned according to the manufacturer's instructions.

The raw expression data resulting from microarray were analyzed using the BeadArray package (Dunning *et al*., 2007) of Bioconductor within the r environment (R Development Core Team 2007). All hybridizations passed the quality control. The data were background‐corrected and normalized with the probe level quantile method. Differential expression analysis was performed with the Limma package (Smyth *et al*., 2005) on intensities that were variance‐stabilized by logarithmic transformation. Annotation provided by Bioconductor was used (illuminaHumanv4BeadID.db). To identify significantly perturbed pathways, we performed SPIA (Tarca *et al*., 2009) analysis on KEGG pathways: genes with *P*‐values < 0.05 were considered to be differentially transcribed. RNA‐seq data have been deposited in the ArrayExpress database at EMBL‐EBI (www.ebi.ac.uk/arrayexpress) under accession number E‐MTAB‐6062.

### 2.15 Proteomic analysis

DU145 cells for proteomic analysis were cultivated in ‘heavy stable isotope labelling with amino acids in cell culture (SILAC)’ low glucose medium (Sigma, D9443) supplemented with l‐arginine‐13C6,15N4 (84 mg·mL^−1^), l‐lysine 13C6 (146 mg·mL^−1^), l‐leucine (0.1 mg·mL^−1^), sodium pyruvate (1 mg·mL^−1^), d‐glucose (25 mm) and 10% dialyzed FBS (Sigma, F0392). Ninety‐five percent efficiency of labelling was achieved after 10 population doublings in heavy media. Low‐adherent cells were prepared according to previously published protocol (Kyjacova *et al*., 2015). Cells grown on T150 tissue culture flasks were irradiated with orthovoltage X‐ray instrument T‐200 (Wolf‐Medizintechnik GmbH) using 0.5 Gy·min^−1^ dose rate and thorium filter for 10 days with 2 Gy (fIR). Every third day, medium was collected and centrifuged. Pelleted cells were resuspended in fresh medium and returned to the original dish. After the last dose, the medium containing low‐adherent cells was transferred to a fresh flask and incubated for additional 24 h to allow the mitotic cells to re‐adhere. Medium was centrifuged at 300 ***g*** for 10 min to pellet the low‐adherent cells. Annexin V‐negative fraction was obtained by incubation with Dead Cell Removal MicroBeads (Dead Cell Removal Kit; Miltenyi Biotec) for 15 min and separation in magnetic field of AutoMACS Pro magnetic separator (Miltenyi Biotec).

Cells from three separations were lysed in lysis buffer (100 mm 4‐ethylmorpholine pH 8.3, 10% acetonitrile, 1% w/v sodium deoxycholate). Lysates were heated to 95 °C for 5 min and sonicated until clear, non‐viscous solution was obtained. Total protein concentration was measured by BCA assay (Thermo, cat No 23225). Lysates from heavy labelled cells (floating population) and nonlabelled cells (adherent non‐irradiated cells) were mixed in a 1 : 1 ratio to obtain a total of 60 μg of protein. Samples were reduced with neutralized 5 mm TCEP (Thermo, 77720) at 60 °C for 15 min followed by alkylation of cysteines with 10 mm MMTS, at room temperature for 30 min. Proteins were digested with trypsin GOLD (Promega, V5280) at 1 : 40 ratio (enzyme : protein) at 37 °C for 16 h.

Digested proteins were acidified by addition of 4 μL of 40% TFA. Precipitated deoxycholate was extracted five times with ethyl acetate. Insoluble particles were spun at 16 000 ***g*** for 15 min and the supernatant was dried using a speed vac.

Peptides were desalted using Empore C18 SPE cartridges. Peptide samples were fractionated offline using Waters XBridge Peptide BEH C18 (2.1 μm × 150 mm) by a linear gradient of mobile phase (A: 20 mm ammonium formate/2% acetonitrile; B: 20 mm ammonium formate/80% acetonitrile) into 42 fractions, which were then combined into 8 final fractions for analysis. Five microliters from each pooled fraction was loaded on Acclaim PepMap 100 nanoViper trap column (C18, 3 μm, 100 Å, 75 μm i.d. × 2 cm; Thermo) for 3 min at a flow rate of 8 μL·min^−1^. Peptides were separated on Acclaim PepMap RSLC column (C18, 2 μm, 100 Å, 75 μm i.d. × 50 cm; Thermo) at a flow rate of 200 nL·min^−1^ and eluted in a 240‐min gradient (A: 2 : 98 acetonitrile/water/0.1% formic acid; B: 80 : 20 acetonitrile/water/0.1% formic acid) and analysed online using a Q Exactive hybrid quadrupole mass spectrometer (Thermo).

Raw data were processed using maxquant (Cox and Mann, 2008). Statistical analysis and annotation enrichment analyses were performed in perseus software (Tyanova *et al*., 2016).

### 2.16 Data processing and statistical analysis

Unless stated otherwise, data are mean values ± standard error of means (SEM) of at least three independent experiments. In mouse experiments, groups of six animals were used, unless stated otherwise. Two‐way ANOVA presented as mean ± SEM was used to assess statistical significance with *P* < 0.05 being regarded as significant, using graphpad prism software, San Diego, CA, USA. Images are representative of at least three independent experiments. Fluorescence‐activated cell sorting (FACS) data were analyzed using flowjo 9.6.4 cytometric analytical software (FlowJo LLC., Ashland, OR, USA).

## 3 Results

### 3.1 5‐Azacytidine induces loss of adhesion and stem cell traits associated with resistance to anoikis

The exposure of human cancer cells to fIR resulted in development of resistant cell subpopulations including cells losing adhesion and bearing CSC‐like features (Kyjacova *et al*., 2015). To find out whether the development of such low‐adherent cell fraction represents a common response of tumour cells to genotoxic stress, we exposed human cancer cell lines HeLa and MCF‐7, and retrovirally immortalized bone marrow mesenchymal stem cells HS‐5 to 5‐azacytidine (5‐AC), a hypomethylating agent currently used in the therapy of high‐risk myelodysplastic syndrome (MDS; Kornblith *et al*., 2002) and tested in numerous clinical trials for treatment of solid malignancies. We used two modes of 5‐AC exposure (as specified for each experiment in the figure legends): (1) a ‘clinical‐mimicking’ regimen of 5‐AC with daily administration for seven consecutive days with cells harvested at day 8 (see Fig. 1A, left), and (2) a single dose of 5‐AC with harvest at day 3 showing a similar effect on resistance development achieved in shorter time (see Fig. 1A, right), both within the concentration range of 5‐AC that is reached in the humans (in MDS patients 5‐AC is administered at a dose of 75 mg·m^−2^·day^−1^; (Marcucci *et al*., 2005; Rudek *et al*., 2005). Both 5‐AC‐treatments mostly reproduced the effects of fIR published previously (Kyjacova *et al*., 2015), showing the equivalence of both 5‐AC treatments. Besides senescence‐like state (not shown) and cell death (see Fig. S1A), 5‐AC treatment induced development of a low‐adherent cell subpopulation (see Fig. S1B), the onset of which was detectable in all three cell types from day 2 onwards (see Fig. 1B). Comparable to fIR, 5‐AC‐induced occurrence of anoikis‐resistant cells that survived in a low‐adherent (dormant) state for 2–4 weeks before readhesion and renewal of proliferation (Fig. S1C; only MCF‐7 cells shown). The low‐adherent cells exhibited stem cell‐like traits such as elevation of SRY (sex determining region Y)‐box 2 (Sox2), CD44 and OCT4 transcripts (see Fig. 1C for HeLa cells and Fig. S1D,E for MCF‐7 and HS‐5 cells), activation of Notch signalling (detected as elevation of Notch ligands DLL1 and DLL4 and Notch effector transcription factors Hes1 and Hey1; see Fig. 1D for HeLa cells and Fig. S1F,G for MCF‐7 and HS‐5 cells) and elevation of Snail (see Figs 1E and S1H for protein and mRNA levels of Snail, respectively). Furthermore, the lack of proliferation in the low‐adherent cell fraction correlated with increased mRNA levels of inhibitors of cyclin‐dependent kinases p16^INK4a^ (p16) and p21^waf1^ (p21; Fig. 1F for HeLa cells and Fig. S1I,J for MCF‐7 and HS‐5 cells; note, the mRNA levels of cell cycle cyclin‐dependent kinases were significantly downregulated as detected by transcriptome analysis). In addition, the level of anti‐apoptotic factor Bcl‐XL was most pronounced in the low‐adherent fraction (Fig. 1G). Together with suppression of protein levels of E‐cadherin and integrin αV (Fig. S1K) this demonstrated that 5‐AC is capable of inducing phenotypic changes reminiscent of those evoked by fIR (Kyjacova *et al*., 2015). The activation of kinases Erk1/2 (detected as phosphothreonine 202 and phosphotyrosine 204 of Erk1 and Erk2, respectively) and Akt (detected as phosphoserine 473) was observed (Fig. S1L). Consistent with a previous study (Kyjacova *et al*., 2015), inhibition of Erk1/2 activity by MEK inhibitor selumetinib suppressed survival of the low‐adherent fraction and reconstitution of fully adherent colonies (Fig. S1M). Of note, siRNA‐mediated knockdown of Bim, a protein involved in anoikis, whose degradation is controlled by Erk activity, resulted in preservation of viability in low‐adherent cells even in the presence of selumetinib (Fig. 1H; for efficiency of Bim downregulation, see Fig. 1I), supporting the role of the Erk/Bim pathway in the survival of low‐adherent cells.

Altogether, these data demonstrate that 5‐AC induces phenotypic plasticity characterized by survival of low‐adherent tumour cells with stem cell‐like features in different human cell types in a manner similar to fIR, thus indicating that this type of cell response is shared by various genotoxic insults.

### 3.2 Elevated interferon response is a common feature of surviving low‐adherent cells

**Figure 2:**
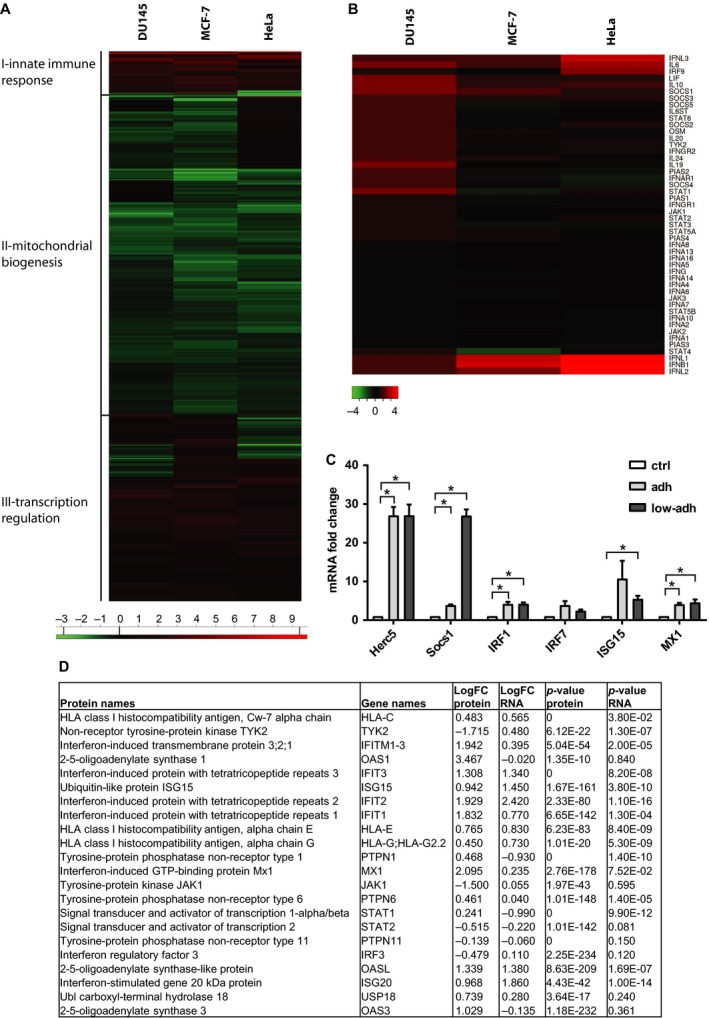
(A) Heat map showing the hierarchical clustering of log2 fold change (LFC) of genes commonly deregulated in all three low‐adherent cell fractions after exposure to stress (DU145 and MCF‐7, 10 × 2 Gy; HeLa, 7 × 4 μm 5‐AC). (B) Heat map showing expression of interferon‐regulated genes selected from cluster I in all three low‐adherent cell fractions after exposure to stress (DU145 and MCF‐7, 10 × 2 Gy; HeLa, 7 × 4 μm 5‐AC). (C) RT‐qPCR validation of changes in the expression of selected genes from microarray analysis in 5‐AC (4 μm)‐treated HeLa cells. Data are shown as mean values ± SEM, with *n *=* *3 per group. Two‐way ANOVA was used for multigroup comparisons followed by Tukey's *post hoc* test. The asterisk represents *P* < 0.05. (D) Correlation of mRNA and protein expression of selected interferon targets in irradiated (10 × 2 Gy) DU145 non‐adherent cells compared with control cells based on annotations.

To characterize further the low‐adherent cells surviving 5‐AC and radiation treatments, a comparative analysis of gene expression profiles of irradiated cells (DU145 and MCF‐7; 10 × 2 Gy) with 5‐AC‐treated cells (HeLa; 7 × daily dose of 4 μm 5‐AC) was performed using Illumina human gene expression array. The hierarchical clustering of resistant populations revealed 2449 commonly deregulated protein‐coding genes in low‐adherent survivors (Fig. 2A, Table S1A). The heat map representation of the clustered matrix showed enrichment of genes involved in the innate immune response pathways (cluster I), specifically, nuclear factor κB (NF‐κB), Toll‐like receptor, and interferon signalling (for details, see Figs 2B and S2A, Table S1B). The changes of mRNA levels of several interferon stimulated genes (*IRF1, IRF7, ISG15, Herc5, SOCS‐1* and *MX‐1*) were validated by RT‐qPCR in HeLa cells treated with 5‐AC for 7 days (Fig. 2C). Cluster II predominantly contained genes involved in the mitochondrial biogenesis and maintenance. Finally, cluster III contained genes involved in the regulation of transcription. These genes were, conversely, upregulated in all stress‐induced low‐adherent cells in comparison with the non‐treated controls (Table S1B).

To obtain a more comprehensive view of the biological state of low‐adherent cells, we performed proteomic profiling of irradiated (10 × 2 Gy) DU145 cells using SILAC and mass spectrometry (Ong *et al*., 2002; Table S2A). As a next step, the protein expression data were correlated with mRNA expression profiles of fIR low‐adherent DU145 cells using 2D annotation enrichment (Cox and Mann, 2012; Fig. S2B, Table S2B). Analogous to gene expression profiles, the proteomic pattern in the fIR low‐adherent DU145 cells was dominated by the ‘interferon response’ pathway including proteins encoded by interferon stimulated genes such as *HLA‐C, OAS1* and *OAS3, ISG15* and *ISG20, MX1* and *STAT1* (Der *et al*., 1998; Fig. S2C; for relative expressions of genes containing this annotation see Fig. 2D).

To conclude, both fIR‐ and 5‐AC‐treatment induced development of the low‐adherent surviving cell fraction characterized by profound changes in the expression of genes and proteins involved mainly in interferon response and cell cycle regulation.

### 3.3 IFNγ but not IFNβ induces loss of adhesion and anoikis‐resistant survival of human cancer cells

**Figure 3:**
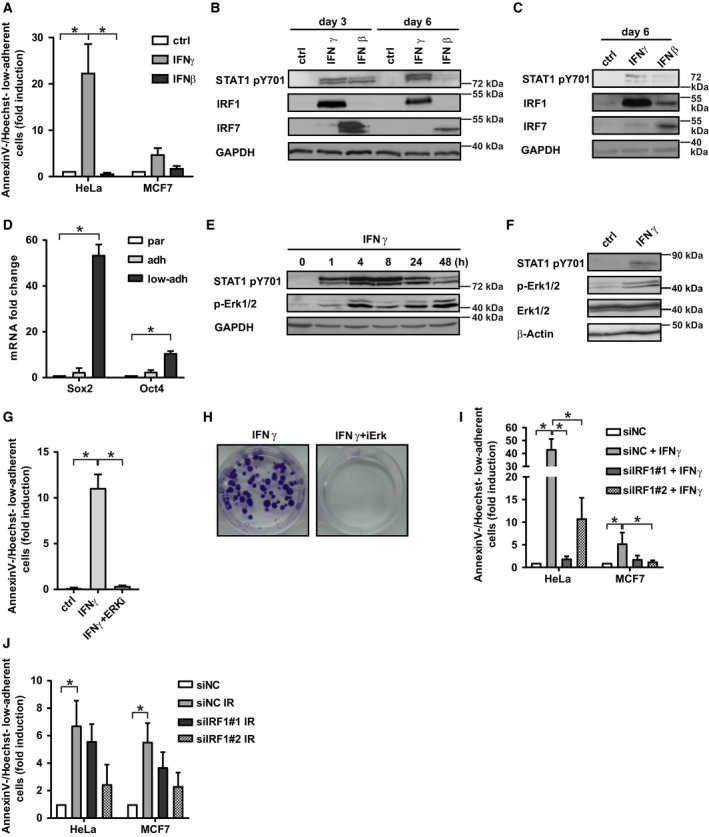
(A) Loss of adhesion expressed as ratio (in fold induction) of Annexin V^−^/Hoechst^−^ detached cells relative to all Annexin V^−^/Hoechst^−^ cells assessed by FACS in HeLa and MCF‐7 cells treated with IFNγ (5 ng·mL^−1^) or IFNβ (50 ng·mL^−1^) for 3 days (HeLa) or 6 days (MCF7). Non‐treated cells were used as a control. Immunoblotting detection of STAT1 phosphorylated on tyrosine 701, IRF1 and IRF7 in control and IFNγ‐ or IFNβ‐treated HeLa (B) or MCF‐7 (C) cells for 3 or 6 days. GAPDH was used as a loading control. (D) RT‐qPCR quantification of *Sox2* and *Oct4* mRNA levels in parental, adherent and low‐adherent HeLa cells treated with IFNγ for 72 h. (E) Immunoblotting detection of STAT1 phosphorylated on tyrosine 701 and Erk phosphorylated on threonine 202/tyrosine 204 in HeLa cells treated with IFNγ for the period as indicated. GAPDH was used as a loading control. (F) Immunoblotting detection of STAT1 phosphorylated on tyrosine 701 and Erk phosphorylated on threonine 202/tyrosine 204 in MCF‐7 cells treated with IFNγ for 72 h. GAPDH was used as a loading control. (G) Estimation of low‐adherent survival after inhibition of Erk kinase with selumetinib (ERKi; 1 μm) expressed as indicated (see above) in HeLa cells treated with IFNγ for 72 h. (H) Clonogenic cell survival assay of HeLa cells treated with IFNγ alone or in combination with Erk inhibition with selumetinib (ERKi; 1 μm) for 72 h and assayed at day 29 after the end of the treatment. (I) Effect of siRNA knockdown of *IRF1* (siIRF1) on the loss of adhesion expressed as indicated (see above) in HeLa and MCF‐7 cells treated with IFNγ for 72 h. Non‐targeting siRNA (siNC) was used as a control. (J) Effect of siRNA knockdown of *IRF1* (siIRF1) on the loss of adhesion expressed as indicated (see above) in DU145 and MCF‐7 cells irradiated with one dose of 10 Gy for 72 h. Non‐targeting siRNA (siNC) was used as a control. Data are shown as mean values ± SEM, with *n *=* *3 per group. Two‐way ANOVA was used for multigroup comparisons followed by Tukey's *post hoc* test. The asterisk represents *P* < 0.05.

Based on the transcriptome and proteomic profiling, we next tested whether the activity of the interferon pathway is causally involved in the development of low‐adherent state and survival of cancer cells. For this purpose, HeLa and MCF‐7 cells were exposed to daily administration of interferon type I (IFNβ) and type II (IFNγ) for 3 or 6 days, and the development of the low‐adherent cell subpopulation was followed. In contrast to IFNβ, IFNγ significantly induced low‐adherent population from day 3 in HeLa and day 6 in MCF‐7 cells (Fig. 3A). Notably, the administration of IFNγ induced the loss of adhesion in HS‐5 cells as well (Fig. S3A). As expected, besides STAT1 receptor‐mediated phosphorylation (detected as STAT1 tyrosine 701 phosphorylation), the response to IFNβ and IFNγ was reflected by induction of transcription factors involved in the late interferon response *IRF7* and *IRF1*, respectively (see Fig. 3B for HeLa and 3c for MCF‐7 cells).

Formation of large extracellular vesicles (see Fig. S3B; Rak, 2010) in both HeLa and MCF‐7 cells was associated with a switch to the amoeboid‐like form of cell migration (Di Vizio *et al*., 2009) of low‐adherent cells (as shown in Video S1). Interestingly, some IFNγ‐induced low‐adherent cells were able to re‐adhere within several days after the end of the treatment and to reestablish adherent cell growth (Fig. 3H, left). Similarly to fIR, the extended exposure of cells to IFNγ resulted in marked elevation of Sox2 and Oct4 mRNA levels in the low‐adherent cell fraction (Fig. 3D; HeLa cells shown) and activation of Erk1/2 (see Fig. 3E for HeLa and Fig. 3F for MCF‐7 cells). Intriguingly, MEK inhibitor selumetinib (Fig. S3C) suppressed the IFNγ‐induced development of low‐adherent cells (Fig. 3G) as well as the establishment of re‐adherence (see Fig. 3H, right), indicating crosstalk between IFNγ and MEK/Erk signalling pathways in the anoikis‐resistant cancer cell survival. Downregulation of late interferon response transcription factor *IRF1* by RNA interference (Fig. S3D,E) resulted in suppression of the IFNγ‐induced (Fig. 3I) as well as IR‐induced (Fig. 3J) low‐adherent cell fraction, supporting the causal role of IFNγ signalling in low‐adherent survival after genotoxic stress.

These data show that prolonged activation of the IFNγ/JAK signalling pathway in human cancer cell models grown *in vitro* induces changes in cell adhesive properties and development of the low‐adherent cell subpopulation matching phenotypic reprogramming observed after fIR and 5‐AC administration, respectively. Altogether, these findings indicate that the development of radioresistant cell subpopulations is mediated by the concerted action of the IFNγ/JAK and Erk signalling pathways.

### 3.4 Upregulation of suprabasin is a component of the shared response to stress induced by 5‐azacytidine, fIR and IFNγ

**Figure 4:**
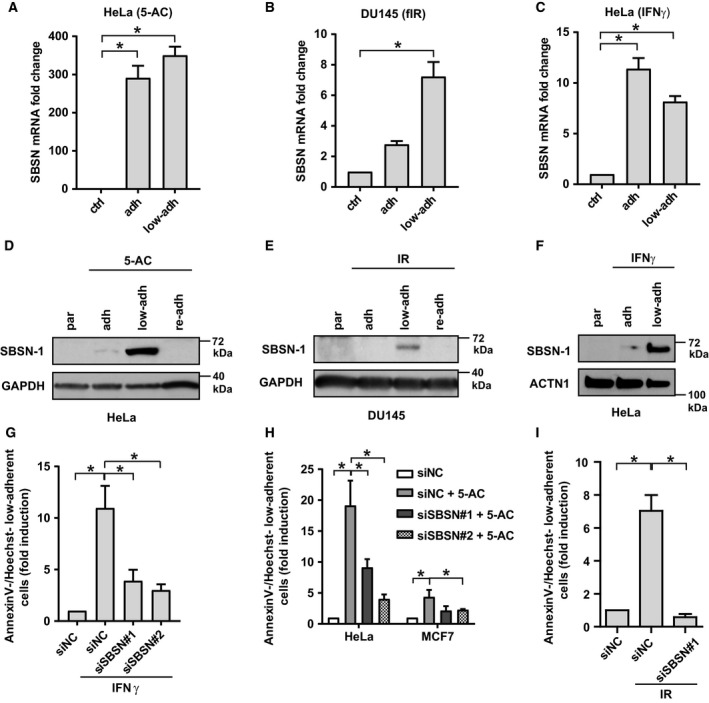
RT‐qPCR quantification of SBSN mRNA level in parental, adherent and low‐adherent HeLa cells treated for 7 days with 4 μm 5‐AC (A), DU145 cells irradiated (fIR) daily with 2 Gy for 10 days (B) and HeLa cells treated with IFNγ (5 ng·mL^−1^) for 72 h (C). Immunoblotting detection of SBSN in parental, adherent and low‐adherent HeLa cells treated with 4 μm 5‐AC for 5 days (D), DU145 cells irradiated (fIR) daily with 2 Gy for 10 days (E) and HeLa cells treated with IFNγ for 72 h (F). GAPDH and ACTN1 were used as loading controls. (G) Effect of siRNA knockdown of *SBSN* (siSBSN) on the loss of adhesion expressed as ratio (in fold induction) of Annexin V^−^/Hoechst^−^ detached cells relative to all Annexin V^−^/Hoechst^−^ cells assessed by FACS in HeLa cells treated with IFNγ for 72 h. Non‐targeting siRNA (siNC) was used as a control. (H) Effect of siRNA knockdown of *SBSN* (siSBSN) on the loss of adhesion expressed as ratio (in fold induction) of Annexin V^−^/Hoechst^−^ detached cells relative to all Annexin V^−^/Hoechst^−^ cells assessed by FACS in HeLa and MCF‐7 cells treated with 2 μm 5‐AC for 72 h. Non‐targeting siRNA (siNC) was used as a control. (I) FACS analysis of the effect of *SBSN* siRNA‐mediated (siSBSN) downregulation in U373 cells on the mobilization of the low‐adherent cell subpopulation after 2 Gy irradiation. The amount of low‐adherent cells is expressed as a ratio (fold induction) of Annexin V^−^/Hoechst^−^ detached cells relative to all Annexin V^−^/Hoechst^−^ cells. Non‐targeting siRNA (siNC) was used as a control. Data are shown as mean values ± SEM, with *n *=* *3 per group. Two‐way ANOVA was used for multigroup comparisons followed by Tukey's *post hoc* test. The asterisk represents *P* < 0.05.

Furthermore, comparative analysis of transcript profiles of low‐adherent cells induced by fIR (DU145 and MCF‐7) and 5‐AC (HeLa) revealed 463 genes whose transcripts were altered in the same direction (see Venn diagram in Fig. S4A). Notably, among the top scoring genes upregulated in surviving low‐adherent cell fractions was *SBSN* (Fig. S4B), an oncoprotein associated with tumour progression and metastasis (Glazer *et al*., 2009; Shao *et al*., 2012; Zhu *et al*., 2016). Indeed, the elevation of *SBSN* transcript levels was confirmed independently by RT‐qPCR in adherent and low‐adherent fractions of all tested models including IFNγ‐treated HeLa cells (Fig. 4A–C). Notably, the protein levels of *SBSN* isoform 1 (61 kDa) were specifically markedly elevated in low‐adherent fractions (see Fig. 4D–F). Intriguingly, RNA interference‐mediated downregulation of *SBSN* (Fig. S4C,D) suppressed the development of low‐adherent cell fraction induced by both IFNγ (Fig. 4G; HeLa cells shown) and 5‐AC (Fig. 4H; HeLa and MCF‐7 cells shown).

As *SBSN* expression is associated with some human malignancies, we searched for cancer cell lines expressing the *SBSN* under unperturbed conditions. *SBSN* expression was not detected in most cell types tested (DU145, LNCaP, 22RV1, PC‐3, MCF‐10a, MCF‐7, MDA‐MB‐231, BT474, ZR‐75‐30, MRC‐5, H1299, HS913T, NCI‐H28, NCM 460, HCT166, HT29, A375, HS‐5, HEK293T, Panc‐1, U2OS, HeLa, hTert RPE‐1, IMR90 and BJ cells; not shown); however, the *SBSN* isoforms 2 and 3 protein levels were detected in cell lysates and conditioned media of human glioblastoma U373 and ovarian carcinoma SK‐OV‐3 cell lines (see Fig. S4E,F for immunoblotting and Fig. S4G,H for indirect immunofluorescence).

To examine further the functional role of *SBSN* in radioresistance, the effect of *SBSN* downregulation on radiosensitivity of the U373 cell line was estimated. Indeed, knockdown of *SBSN* suppressed the development of IR‐induced (2 Gy) low‐adherent cell fraction (Fig. 4I) and increased the percentage of apoptotic cells (Fig. 4I), supporting the functional role of *SBSN* in radioresistance of glioblastoma cells.

Altogether, our findings show that the expression of *SBSN* is a shared feature of the cell response to genotoxic stress induced by different stimuli and indicate that *SBSN* is required for the stress‐induced phenotypic plasticity, cellular heterogeneity and resistance to apoptosis of tumour cell populations.

### 3.5 IFNγ‐ and 5‐AC‐induced expression of *SBSN* is mediated by the MEK/Erk pathway

**Figure 5:**
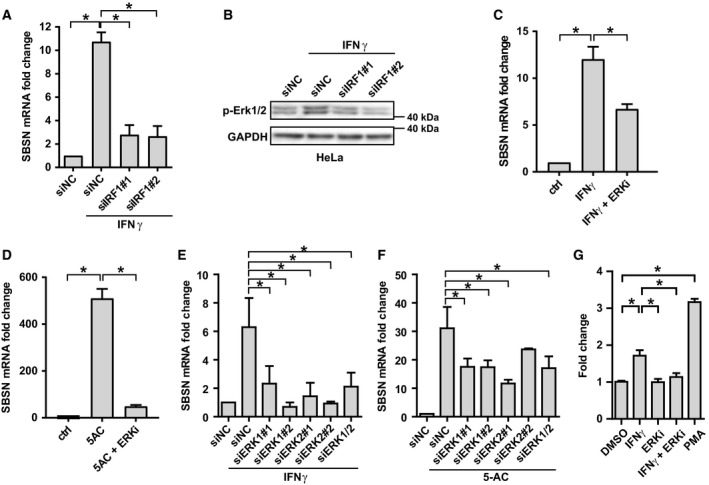
(A) Effect of siRNA knockdown of IRF1 (siIRF1) on the expression of *SBSN* gene detected by RT‐qPCR in HeLa cells treated with IFNγ (5 ng·mL^−1^) for 72 h. Non‐targeting siRNA (siNC) was used as a control. (B) Effect of siRNA knockdown of *IRF1* (siIRF1) on Erk phosphorylated on threonine 202/tyrosine 204 detected by immunoblotting in HeLa cells treated with IFNγ for 72 h. GAPDH was used as a loading control. Effect of Erk kinase inhibition by selumetinib (ERKi; 1 μm) on the *SBSN* expression assessed by RT‐qPCR in HeLa cells treated with IFNγ (5 ng·mL^−1^) (C) or 2 μm 5‐AC (D) for 72 h. Effect of siRNA knockdown of *Erk1* (siErk1) or *Erk2* (siErk2) on the SBSN expression assessed by RT‐qPCR in HeLa cells treated with IFNγ (E) or 2 μm 5‐AC (F) for 72 h. Non‐targeting siRNA (siNC) was used as a control. (G) *SBSN* proximal promoter‐driven *Firefly* luciferase activity in HEK293 cells exposed to IFNγ (5 ng·mL^−1^), MEK/Erk inhibitor selumetinib (ERKi; 1 μm), their combination and PMA (20 nm) for 48 h. *SBSN* luciferase activity for each condition is expressed relative to promoterless pGL3‐Basic construct. Data are shown as mean values ± SEM, with *n *=* *3 per group. Two‐way ANOVA was used for multigroup comparisons followed by Tukey's *post hoc* test. The asterisk represents *P* < 0.05.

Next we examined how the *SBSN* gene is regulated by IFNγ and other stresses. As shown in Fig. 5A, RNA interference‐mediated knockdown of *IRF1* resulted in suppression of IFNγ‐induced elevation of the *SBSN* transcript in HeLa cells. As IFNγ activated Erk1/2 (Fig. 3E,F) and MEK inhibitors affected IFNγ‐induced development of low‐adherent fraction (Fig. 3G), we examined whether *SBSN* is regulated by IFNγ‐induced activation of Erk signalling. Downregulation of IRF1 suppressed IFNγ‐induced activity of Erk1/2 (Fig. 5B) and treatment of IFNγ‐exposed HeLa cells with selumetinib resulted in suppression of the IFNγ‐induced upregulation of *SBSN* (Fig. 5C) to a degree comparable to the impact of Erk1/2 downregulation (Fig. 5E; see Fig. S5A for efficiency of Erk1/2 downregulation), indicating that Erk activity contributes to the observed upregulation of *SBSN* gene expression mediated by IFNγ. The effect of inhibition of the Erk pathway (Fig. S5B) on the mRNA expression of *SBSN* was also found in HeLa and MCF‐7 cells exposed to both 5‐AC and selumetinib (Figs 5D and S5C,D; note MEK inhibitor U0126 was used to further support the data). Additionally, the downregulation of Erk1 and/or Erk2 also suppressed 5‐AC‐induced elevation of *SBSN* mRNA (see Fig. 5F; for Erk1/2 downregulation efficiency, see Fig. S5E), suggesting again the role of the Erk pathway in stress‐induced *SBSN* expression.

Furthermore, a luciferase activity driven by the proximal regulatory region of the *SBSN* gene spanning nucleotides −2000 to +71 relative to the *SBSN* transcription start site (pGL3‐*SBSN*) was estimated in HEK293 cells treated with either IFNγ or MEK inhibitor, or both (see Section 2, Materials and methods). Indeed, IFNγ treatment significantly induced *Firefly* luciferase expression driven by the *SBSN* proximal promoter relative to a promoterless construct (pGL3‐Basic), whereas the MEK inhibitor selumetinib almost completely abolished it (Fig. 5G), suggesting the effect of IFNγ on *SBSN* gene expression is mediated by Erk1/2 signalling. Moreover, an activator of MAPK signalling PMA induced *SBSN* promoter‐driven *Firefly* luciferase expression, further supporting transcriptional control of *SBSN* by Erk1/2 in accordance with a previous study (Park *et al*., 2002).

Collectively, these findings indicate the crucial role of Erk kinase signalling in the upregulation of *SBSN* expression in cells exposed to genotoxic stress.

### 3.6 *SBSN* is expressed in malignant human tissues and is associated with metastasis

**Figure 6:**
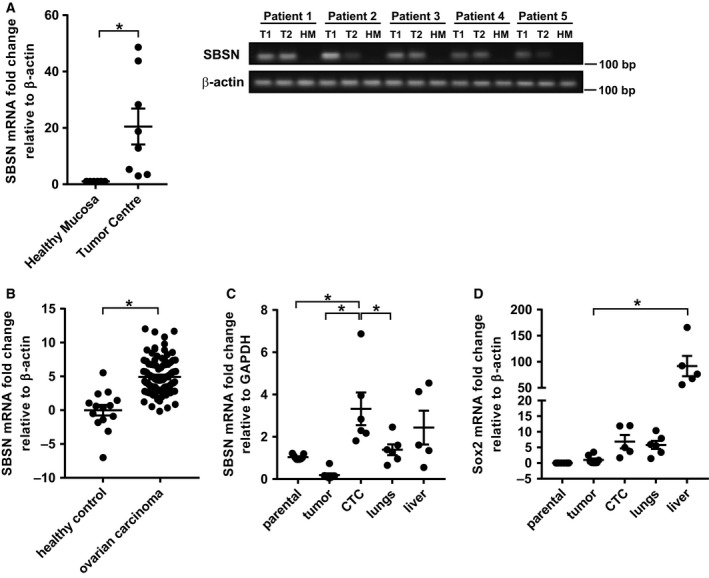
(A) RT‐qPCR quantification of *SBSN* mRNA level in human colorectal tumours. Two samples from two different tumour sites were analysed (T1, T2; for statistic evaluation, values from T1 and T2 were averaged) and compared with healthy surrounding mucosa (HM), with *n *=* *8 per group. (B) RT‐qPCR quantification of *SBSN* mRNA level in human ovarian cancer samples, with *n *=* *14 for healthy controls and *n *=* *100 for ovarian carcinoma samples. RT‐qPCR quantification of SBSN (C) and Sox2 (D) mRNA level in 4T1 parental cells (grown *in vitro*), 4T1 cells forming solid tumour, 4T1 cells circulating in blood (CTC) and 4T1 metastasis in lungs and liver of BALB/c mice, with *n *=* *5–6 per group. Data are shown as mean values ± SEM. Two‐way ANOVA was used for multigroup comparisons followed by Tukey's *post hoc* test. The asterisk represents *P* < 0.05.

Recent reports indicate *SBSN* expression in human malignancies such as glioblastoma (Formolo *et al*., 2011) or lung cancer (Glazer *et al*., 2009). Indeed, the analysis of *SBSN* expression in colorectal cancer biopsies (*n* = 8) provided similar results (Fig. 6A). While normal mucosa surrounding the tumour tissue was always negative for *SBSN* mRNA expression, all tumour samples were positive (*P *<* *0.05). Similarly, the *SBSN* mRNA levels in ovarian carcinoma biopsies (Fig. 6B; *n* = 100) were significantly higher than in non‐malignant ovary tissue (*n* = 14, *P *<* *0.0001).

Due to association of *SBSN* expression with EMT programme and cell stemness observed in our *in vitro* experiments, we next examined whether the *SBSN* expression is associated with development of metastases *in vivo*. Using a mouse model of 6‐thioguanine‐resistant 4T1 cells that frequently metastasize to lungs and liver, we compared *SBSN* transcript levels in parental cells, primary tumours, 4T1‐derived circulating tumour cells (CTCs), and lung and liver metastases (see Section 2 Materials and methods for details). Notably, the level of *SBSN* expression was significantly higher in CTC and lung and liver metastases than in primary tumour and parental 4T1 cells (Fig. 6C), a feature that correlated with increased Sox2 expression in these cells (Fig. 6D).

Altogether, *SBSN* is expressed in fully developed human malignant lesions of colorectal and ovarian carcinomas. Furthermore, the high expression of *SBSN* in circulating tumour cells and metastatic lesions detected in our mouse model suggests association of *SBSN* with the metastatic process.

## 4 Discussion

Metastatic dissemination frequently combined with development of resistance to current antitumour therapies represents the most serious complications of malignant diseases and causes more than 90% of deaths among cancer patients. The aim of this study was to elucidate the mechanisms of acquired resistance to radiotherapy and chemotherapy in several human cancer cell models, with emphasis on better understanding the development of a subpopulation of cancer cells characterized by anoikis‐resistant survival. Among many deregulated genes detected by cDNA microarray analysis of transcript profiles from anoikis‐resistant human tumour cells induced by 5‐azacytidine or fIR, we identified elevation of gene transcripts regulated by the interferon signalling pathway and IFNγ itself (but not IFNβ) as being capable of inducing the anoikis‐resistant state. Furthermore, we pinpointed *SBSN*, the recently reported oncoprotein, as an effector factor involved in the development of cancer cell resistance phenotypes. Elevated expression of *SBSN* in mouse circulating tumour cells and metastases, as well as clinical samples of human colorectal and ovarian carcinomas, supported our *in vitro* findings that *SBSN* was involved in cancer development and progression.

Interferons are known to induce genotoxic stress (see Hubackova *et al*., 2015 and references therein). Here we observed that the exposure of tumour cells to IFNγ but not IFNβ was sufficient to mobilize a heterogeneous low‐adherent and floating matter composed mainly of apoptotic and necrotic cell bodies, and a small fraction of cells that survived the IFNγ‐induced stress and later re‐established adherent cell growth. These results indicate that interferon response itself or responses triggered by genotoxic insults alter cell adhesion properties in a subpopulation of cancer cells that is also associated with resistance to anoikis resulting in increased invasiveness of these cells.

As was shown previously (Kyjacova *et al*., 2015) low‐adherent cells with stem cell‐like features are continuously generated from the adherent, senescent phenotype‐developing fraction of resistant cells. Although the low‐adherent cells partially share their gene expression pattern with senescent cells, for example elevated p21^waf1^ and p16^INK4a^, and several SASP factors, our comparison of global transcription profiles of low‐adherent versus adherent cells indicates they are not identical. The major functional difference between the low‐adherent and adherent cell fraction is evident from the ability of the former cells to re‐attach and re‐enter the cell cycle after a few weeks. This correlates with the recently published findings that a reprogrammed gene expression profile associated with de‐differentiation, stem cell‐like features, EMT, increased levels of c‐myc and STAT, which we observed also in our low‐adherent cells, is responsible for senescence bypass and therapy resistance (Deschênes‐Simard *et al*., 2019). Furthermore, given that cytoplasmic DNA elicits IFN responses (primarily type I IFNβ) and NF‐κB signalling through the cGAS/STING pathway (Sun *et al*., 2013), we also searched the transcriptomes of the anoikis‐resistant cell subpopulation for evidence of IFNβ and NF‐κB activity. Indeed, our data show increased mRNA levels of IFNB1, IL1, tumour necrosis factor (TNF), IL6 and IL18, common targets of the cGAS/STING pathway. Future studies should address the possibility that the cGAS/STING pathway may contribute to development of the low‐adherent phenotype and thereby play a role in survival and radiotherapy‐resistance of cancer cells.

Our analysis of IFNγ‐induced elevation of *SBSN* revealed that activation of Erk1/2 is a prerequisite for *SBSN* expression reminiscent of endothelial cells treated with epidermal growth factor as an activator of Erk pathway (Alam *et al*., 2014). Treatment of cells with IFNγ resulted in increased Erk1/2 activity, the inhibition of which by MEK inhibitors reduced both *SBSN* expression and occurrence of low‐adherent population after fIR, 5‐AC or IFNγ. Although we did not explore the mechanism of Erk activation by IFNγ in depth, several reports show the ability of IFNγ to activate the Erk pathway (see Cho *et al*., 2007; Halfter *et al*., 2005; Harris *et al*., 2007; Sakatsume *et al*., 1998; Stancato *et al*., 1998). Upon IFNγ stimulation of keratinocytes, *SOCS1* induced by JAK/STAT pathway inhibited IFNγ receptor and STAT1 phosphorylation but maintained Erk activation (Madonna *et al*., 2008). Together with our observation of increased expression of *SOCS1* in the low‐adherent cells (Fig. 2C), these results implicate *SOCS1* in modulation of therapy resistance via stabilization of Erk signalling.

Although the exact molecular function of *SBSN* remains currently unknown, there is emerging evidence that aberrant expression of *SBSN* is associated with human malignancies. *SBSN* has been found among genes coordinately expressed in non‐small cell lung carcinoma characterized by promoter hypomethylation (Glazer *et al*., 2009). Intriguingly, ectopic expression of *SBSN* in some lung squamous cell carcinoma cell lines and normal lung fibroblasts is associated with growth advantage (Glazer *et al*., 2009). Likewise, *SBSN* promotes proliferation of oesophageal squamous cell carcinoma (ESCC) cell lines *in vitro* (Zhu *et al*., 2016). In addition, *SBSN* expression correlated with ESCC progression (i.e. *SBSN* expression correlated positively with increasing clinical tumour staging and negatively with overall patient survival time; Zhu *et al*., 2016). Furthermore, *SBSN* was found as part of the secretome of highly invasive glioblastoma cell lines (Formolo *et al*., 2011). Notably, aberrant expression of *SBSN* was found in pleural effusions of lung adenocarcinoma patients (Sheng and Zhu, 2014) and in tumour endothelial cells of renal and colon carcinomas (Alam *et al*., 2014). These recent findings of cancer‐associated expression of *SBSN* were further supported and extended in our present study by detection of *SBSN* in colorectal and ovarian carcinomas.

The presence of *SBSN* transcript in both adherent and anoikis‐resistant low‐adherent cell populations, yet present predominantly in the latter cell fraction, suggested a potentially novel role of *SBSN* in the development of low‐adherent state and anoikis‐resistant survival. This scenario was indeed supported by the experiments manipulating *SBSN* levels, where the expression of *SBSN* dictated the amount of cells in the low‐adherent state. *SBSN* overexpression activates the Wnt/β‐catenin signalling pathway (Zhu *et al*., 2016), which has been implicated as a niche factor to maintain stem cells in a self‐renewing state (Nusse, 2008). Wnt/β‐catenin signalling is often associated with EMT as an activator of EMT transcription factors Zeb, Snail or Slug (Medici *et al*., 2008; Sanchez‐Tillo *et al*., 2011). Thus, expression of *SBSN* in low‐adherent cells can be responsible both for EMT due to increased expression of *Snail* and *Zeb2* (Figs 1E and S1G), and for induction of cancer stemness (Figs 1C and S1C,D).

Based on these findings we propose the role of *SBSN* in stress resistance of cancer cells. It should be emphasized that *SBSN* may be involved in the maintenance of tumour invasiveness and metastatic potential, as was reported for salivary gland adenoid cystic carcinoma (Shao *et al*., 2012). The association of *SBSN* expression with tumour aggressiveness is underscored by our data indicating that *SBSN* induction is highest in circulating mouse tumour cells and metastatic deposits. Collectively, these findings implicate *SBSN* in promoting the aggressive phenotype of tumour cells.

Notably, Aibara *et al*. (2018) identified anti‐*SBSN* antigen activity in immune complexes present in cerebrospinal fluid of patients with the neuropsychiatric form of systemic lupus erythematosus. This example of *SBSN* immunogenicity, documented by autoantibodies reactive with astrocytes causing an inflammatory response, may have implications for future attempts to apply immunotherapy against *SBSN*‐expressing human malignancies. Consistent with the presence of *SBSN* expression in brains of newborn mice (Park *et al*., 2002), our data indicate that *SBSN* can be expressed under certain conditions in cells other than the suprabasal layer of the epidermis. From this perspective, *SBSN* may serve as a tumour neo‐antigen to be exploited in anti‐cancer immunotherapy, particularly for tumours resistant to standard‐of‐care treatment modalities such as radiation used in our present study.

## 5 Conclusions

In conclusion, our findings indicate that genotoxic stresses accompanying radiotherapy or chemotherapy can induce plastic changes of the cancer cell phenotype and increased heterogeneity of surviving cancer cell populations. We showed that survival of a subset of cancer cells in a low‐adherent state depends on the concerted activity of IFNγ and Erk signalling pathways activated by radiation‐ or chemically induced stress (Fig. 5G). Furthermore, the expression of *SBSN* in such low‐adherent compartments of radio‐ and chemo‐resistant cells suggests an unorthodox mechanism through which cancer cells can respond to genotoxic stress. Notably, our data highlight the dependency on *SBSN* as a potential vulnerability of aggressive and metastatic tumours that are commonly refractory to current standard‐of‐care treatments.

## Author contributions

The authors declare no conflict interest.

## Conflict of interest

We confirm that the data presented in the manuscript are novel, they have not been published, and are not under consideration for publication elsewhere. The authors declare that they have no conflicts of interest.

## Supporting information


**Fig. S1.** Generation of the low‐adherent stem cell‐like cells with 5‐AC. (A) Induction of apoptosis expressed as a percentage of Annexin V^+^/Hoechst^+^ cells assessed by FACS in control or HeLa cells treated with 5‐AC (2 μm) for 72 h. (B) Immunofluorescence detection of DAPI (blue) and autofluorescent signal (green) in low‐adherent HeLa cells treated with 2 μm 5‐AC for 72 h. Scale bar: 10 μm. (C) Phase contrast microscopic images of chemoresistant MCF‐7 cell populations 16 and 24 days after treatment with 0.5 μm 5‐AC. Cells were treated for 7 days, low‐adherent cells were then removed and cultivated in fresh medium. Scale bar: 100 μm. RT‐qPCR quantification of *Sox2* (D), *Hes1, DLL1, DLL4* (F) and p21 and p16 (I) expression in parental, adherent and low‐adherent MCF‐7 cells treated with 2 μm 5‐AC for 72 h. RT‐qPCR quantification of *Sox2* and *OCT4* (E), *Hes1, Hey1, DLL4* (G) and p21 and p16 (J) expression in parental, adherent and low‐adherent HS‐5 cells treated with 2 μm 5‐AC for 72 h. (H) RT‐qPCR quantification of Snail expression in parental, adherent and low‐adherent MCF‐7, HeLa and HS‐5 cells treated with 2 μm 5‐AC for 72 h. (K) Immunoblotting detection of E‐cadherin and ITGAV in parental, adherent and low‐adherent MCF‐7 cells treated with 4 μm 5‐AC for 72 h. GAPDH was used as a loading control. (L) Immunoblotting detection of total and threonine 202/tyrosine 204‐phosphorylated Erk and total and serine 473‐phosphorylated Akt in HeLa and MCF‐7 cells treated with 4 μm 5‐AC for 24 and 48 h. GAPDH was used as a loading control. (M) Clonogenic cell survival assay of HeLa cells treated with 2 μm 5‐AC for 72 h in the presence of MEK/Erk inhibitor selumetinib (ERKi; 1 μm). Surviving re‐adherent HeLa cells were detected on day 24 following treatment. Data are shown as mean values ± SEM, with *n *=* *3 per group. Two‐way ANOVA was used for multigroup comparisons followed by Tukey's *post hoc* test. The asterisk represents *P* < 0.05.

**Fig. S2.** Analysis of proteome and transcriptome of 5‐AC‐ and fIR‐treated cells. (A) Genes in GOBP category: ‘response to type I interferon’ with their logarithm fold change values compared with control. (B) Correlation of mRNA and protein expression in irradiated (10 × 2 Gy) DU145 low‐adherent cells compared with control cells based on annotations. Numbers on the *x‐* or *y*‐axis represent the score achieved in 2D annotation enrichment analysis. Terms with both coordinates positive and negative are upregulated/downregulated concomitantly in the proteome and transcriptome, the rest is regulated discordantly. (C) Table containing the exact terms that have both score values > 0.4 or < −0.4.

**Fig. S3.** IFNγ induced the low‐adherent phenotype. (A) Loss of adhesion expressed as a ratio (fold induction) of Annexin V^−^/Hoechst^−^ detached cells relative to all Annexin V^−^/Hoechst^−^ cells assessed by FACS in control HS‐5 cells or cells treated with IFNγ (5 ng·mL^−1^) for 3 days. (B) Phase contrast microscopic image of MCF‐7 cells irradiated for 6 days with one dose of 10 Gy. Large extracellular vesicles are marked with yellow arrows. Scale bar 10 μm. (C) Immunoblotting detection of STAT1 phosphorylated on tyrosine 701 and Erk1/2 phosphorylated on threonine 202/tyrosine 204 in HeLa cells treated with IFNγ (5 ng·mL^−1^) for 72 h in the presence of MEK/Erk inhibitor selumetinib (ERKi; 1 μm). GAPDH was used as a loading control. (D) Immunoblotting detection of STAT1 phosphorylated on tyrosine 701 and IRF1 in HeLa and MCF‐7 cells treated with IFNγ (5 ng·mL^−1^) for 72 h after knockdown of IRF1 (siIRF1). Non‐targeting siRNA (siNC) was used as a control. GAPDH was used as a loading control. (E) Immunoblotting detection of IRF1 in DU145 and MCF‐7 cells 72 h after irradiation with one dose of 10 Gy after knockdown of *IRF1* (siIRF1). Non‐targeting siRNA (siNC) was used as a control. GAPDH was used as a loading control. Data are shown as mean values ± SEM, with *n *=* *3. Two – tailed unpaired Student's *t*‐test was used for comparison between two groups for statistical significance. The asterisk represents *P* < 0.05.

**Fig. S4.** Transcriptome analysis of cell populations after fIR and 5‐AC exposure and SBSN detection. (A) Venn diagram of upregulated genes in low‐adherent HeLa cells treated with 4 μm 5‐AC for 7 days and MCF‐7 and DU145 irradiated daily with 2 Gy for 10 days. Numbers represent single and common significantly up‐ or downregulated genes in the control (non‐treated) sample. (B) Heat map showing the hierarchical clustering of LFC of genes commonly upregulated in all three low‐adherent cell fractions after exposure to stress (DU145, 10 × 2 Gy; MCF‐7, 10 × 2 Gy, HeLa, 5‐AC). (C) RT‐qPCR quantification of *SBSN* expression in HeLa cells after *SBSN* knockdown (siSBSN) treated with IFNγ (5 ng·mL^−1^) for 72 h. (D) RT‐qPCR quantification of *SBSN* expression in HeLa and MCF‐7 cells with *SBSN* knock down (siSBSN) treated with 4 μm 5‐AC for 72 h. Immunoblotting detection of the *SBSN* isoforms in the cell lysates (E) and conditioned media (F) of the U373 and SK‐OV‐3 cells. The SBSN signal was suppressed by the *SBSN* siRNA (siSBSN; 48 h after the RNAi‐mediated knockdown of the SBSN). Non‐targeting siRNA (siNC) was used as a control. Ponceau S staining was used for a control of protein loading. Immunofluorescence detection of the SBSN in the U373 cells irradiated with a single dose of 2 Gy using LS‐C162878 (G) or HPA067734 (H) antibodies. Nuclei were stained with DAPI (1 μg·mL^−1^). Scale bar: 10 μm. (I) Quantitative FACS analysis of apoptosis using Annexin V/Hoechst staining of non‐irradiated (control) or single‐dose (2 Gy)‐irradiated U373 cells. Non‐targeting siRNA (siNC) was used as a control. Data are shown as mean values ± SEM, with *n *=* *3. Two‐way ANOVA was used for multigroup comparisons followed by Tukey's *post hoc* test. The asterisk represents *P* < 0.05.

**Fig. S5.** Effect of the ERK1/2 pathway on the survival of cells exposed to 5‐AC. (A) Immunoblotting detection of total Erk1/2 in HeLa cells treated with IFNγ (5 ng·mL^−1^) for 72 h after knockdown of *Erk1* (siErk1) or *Erk2* (siErk2). Non‐targeting siRNA (siNC) was used as a control. GAPDH was used as a loading control. (B) Immunoblotting detection of Erk phosphorylated on threonine 202/tyrosine 204 in HeLa cells treated with 2 μm 5‐AC for 72 h in the presence of MEK/Erk inhibitor selumetinib (ERKi; 1 μm). GAPDH was used as a loading control. (C) RT‐qPCR quantification of *SBSN* expression in MCF‐7 cells treated with 2 μm 5‐AC for 72 h in the presence of MEK/Erk inhibitors selumetinib (ERKi; 1 μm) or U0126 (10 μm). (D) Immunoblotting detection of Erk phosphorylated on threonine 202/tyrosine 204 in MCF‐7 cells treated with 2 μm 5‐AC for 72 h in the presence of MEK/Erk inhibitors selumetinib (ERKi; 1 μm) or U0126 (10 μm). GAPDH was used as a loading control. (E) Immunoblotting detection of Erk1 and Erk2 in HeLa cells treated with 2 μm 5‐AC for 72 h after knockdown of Erk1 (siErk1) or Erk2 (siErk2). Non‐targeting siRNA (siNC) was used as a control. GAPDH was used as a loading control. Data are shown as mean values ± SEM, with *n *=* *3 per group. Two‐way ANOVA was used for multigroup comparisons followed by Tukey's *post hoc* test.

**Table S1.** Transcriptome analysis of cells surviving fIR and 5‐AC treatment. Excel table containing the log_2_ fold‐change (log_2_FC) of mRNA expression significantly deregulated in irradiated (10 × 2 Gy) DU145 and MCF‐7 cells and 5‐AC (7 × 4 μm)‐treated HeLa cells compared to non‐irradiated, non‐treated control cells (1A) and results from annotation enrichment performed on clusters from the heat map (1B). *P* – values were adjusted using the Benjamini–Hochberg false discovery rate (FDR) method.

**Table S2.** Changes of functional categories on the proteome and transcriptome level of irradiated low‐adherent DU145 cells. Gene ontology (GO) biological processes (GOBP), molecular functions (GOMF), cellular compartments (GOCC), and KEGG pathways were analyzed with 1D protein (2a) and 2D protein and mRNA annotation enrichment (2b) obtained from the comparison of irradiated (10 × 2 Gy) low‐adherent and non‐irradiated control DU145 cells. *P* – values were adjusted using the Benjamini–Hochberg false discovery rate (FDR) method.

**Table S3.** Description of human colon carcinoma and ovarian cancer samples.

**Video S1.** Video of the amoeboid‐like form of cell migration of low‐adherent cells.
